# A Lipiodol Pickering Emulsion Stabilized by Iron‐Doped Carbon Nanozymes for Liver Transarterial Chemoembolization

**DOI:** 10.1002/advs.202410873

**Published:** 2024-12-10

**Authors:** Xiancheng Xia, Yang Li, Rongkang Huang, Yuanbin Wang, Wenxuan Xiong, Hui Zhou, Min Li, Xidong Lin, Youchen Tang, Bo Zhang

**Affiliations:** ^1^ Department of Interventional Center Biomedical Innovation Center The Sixth Affiliated Hospital Sun Yat‐Sen University Guangzhou 510655 P. R. China; ^2^ The Eighth Affiliated Hospital Sun Yat‐sen University Shenzhen 518033 P. R. China; ^3^ Department of General Surgery (Colorectal Surgery) Guangdong Provincial Key Laboratory of Colorectal and Pelvic Floor Diseases Biomedical Innovation Center The Sixth Affiliated Hospital Sun Yat‐Sen University Guangzhou 510655 P. R. China; ^4^ PCFM Lab School of Chemistry Sun Yat‐sen University Guangzhou 510006 P. R. China; ^5^ Department of Gastrointestinal Surgery The Affiliated Dongguan Songshan Lake Central Hospital Guangdong Medical University Dongguan 523326 P. R. China; ^6^ Future Technology School Shenzhen Technology University Shenzhen 518118 P. R. China

**Keywords:** chemodynamic therapy, chemoembolization, liver cancer, Pickering emulsion

## Abstract

Transarterial chemoembolization (TACE) utilizing a water‐in‐oil lipiodol emulsion is a preferable therapeutic strategy for advanced liver cancer in clinical practice. However, the low stability of the lipiodol emulsion and poor efficacy of chemotherapeutic drug seriously undermine the efficiency of TACE. Herein, a novel lobaplatin‐loaded lipiodol emulsion (denoted as ICN‐LPE) is developed by constructing a lipiodol Pickering emulsion (LPE) stabilized with iron‐doped carbon nanozymes (ICN) to mitigate the issue of lipiodol‐water separation. This novel emulsion not only solves the instability of conventional lipiodol emulsions, but also facilitates the sustained release of lobaplatin. More importantly, upon entry into tumor cells, ICN catalyze the generation of reactive oxygen species via the Fenton‐like reaction while simultaneously consuming intracellular glutathione, thereby inducing tumor cell death via chemodynamic therapy. By integrating chemotherapy and chemodynamic therapy, ICN‐LPE demonstrates a synergistic antitumor effect and effectively inhibits tumor growth in a rabbit liver tumor model. Therefore, our ICN‐LPE shows an appealing clinical application prospect for TACE.

## Graphical abstract

A lipiodol Pickering emulsion is stabilized by iron‐doped carbon nanozymes, which not only permits the sustained release of the chemotherapeutic drug but also causes excessive accumulation of highly toxic ROS within tumor cells through the Fenton‐like reaction and the consumption of GSH to induce tumor cell death, leading to excellent antitumor effect in liver transarterial chemoembolization.

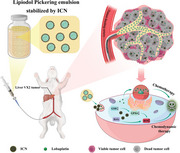

## 1 Introduction

Liver cancer is the sixth most prevalent malignancy worldwide and the third leading cause of cancer‐related death, and its incidence and mortality are continually rising.^[^ ^1^, ^2^ ^]^ Surgical resection, liver transplantation, and local ablation are generally considered as the curative treatments for early‐stage liver cancer.^[^ ^3^, ^4^, ^5^ ^]^ Unfortunately, most patients with liver cancer are diagnosed at an advanced stage and cannot undergo complete surgical resection.^[^ ^6^, ^7^ ^]^ Transarterial chemoembolization (TACE) is usually the main treatment for controlling the progression of the tumor and serves as a transitional treatment for surgical resection.^[^ ^8^, ^9^ ^]^ The principle of TACE is based on the knowledge that liver cancer derives 75% of its blood supply from the hepatic artery.^[^ ^10^ ^]^ By injecting embolic materials carried with chemotherapeutic drugs directly into the hepatic artery, TACE can block the blood supply to limit nutrient provision and facilitate the locally released chemotherapeutic drugs to kill tumor cells, which could improve the therapeutic efficacy while minimizing systemic adverse effects.^[^ ^11^, ^12^ ^]^ However, the low stability of commonly used lipiodol emulsions containing a single chemotherapeutic drug would lead to burst release of the drug and chemoresistance, and the overall 2‐year survival rate of patients after TACE is as low as 31%.^[^ ^13^ ^]^ Therefore, the development of novel injectable embolic materials with excellent antitumor effect plays a vital role in the treatment of liver cancer.

**Figure 1:**
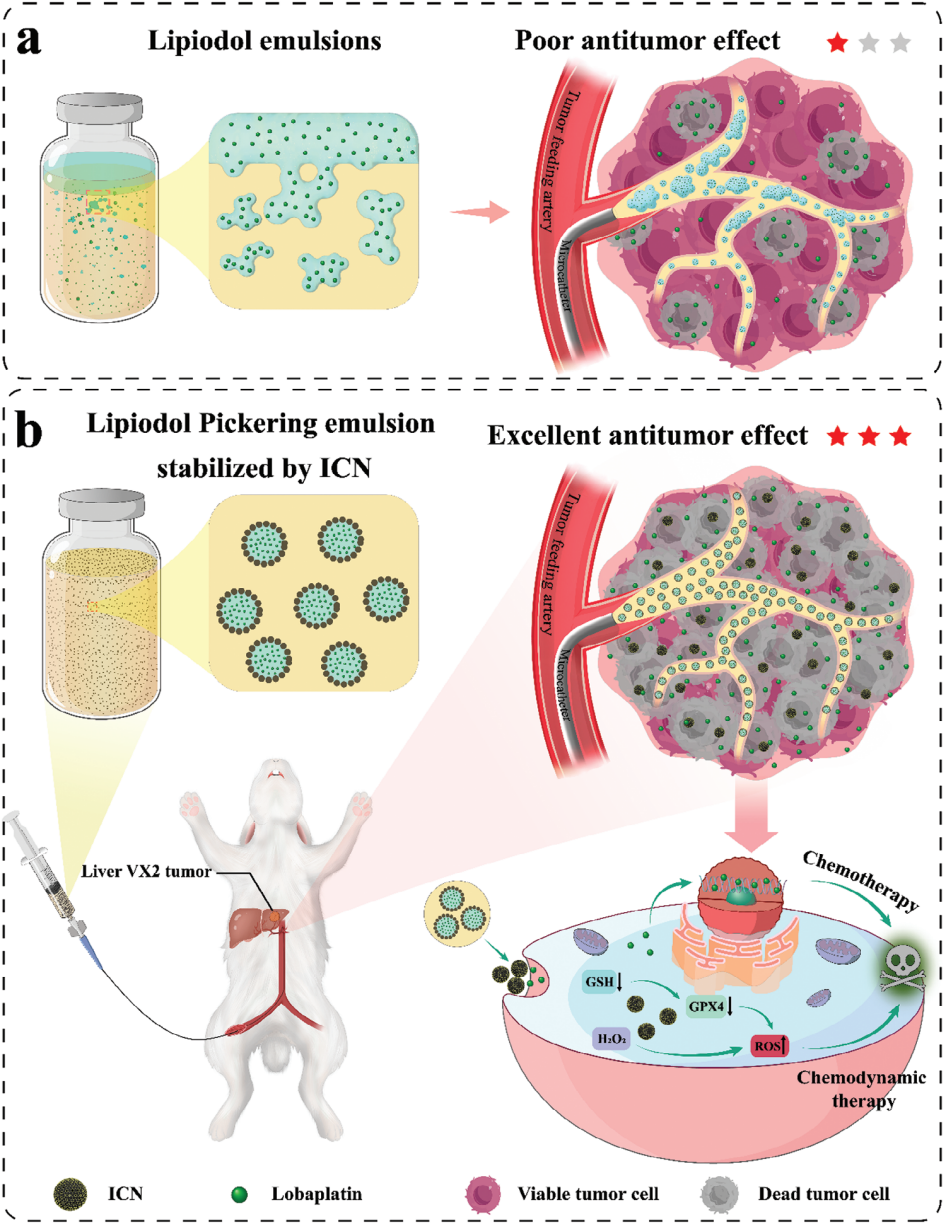
Schematic diagram of a) conventional lipiodol emulsions and b) lipiodol Pickering emulsion stabilized by ICN. Conventional lipiodol emulsions show rapid phase separation, leading to burst release of chemotherapeutic drug and poor antitumor effect. ICN‐LPE not only permits the sustained release of chemotherapeutic drug but also causes excessive accumulation of highly toxic ROS within tumor cells through the Fenton‐like reaction and the consumption of GSH to induce tumor cell death, leading to excellent antitumor effect.

The antitumor mechanism of TACE includes physical embolization and the cytotoxic effect of chemotherapeutic drugs.^[^ ^14^ ^]^ The classic embolic material lipiodol is hydrophobic, while chemotherapeutic drugs (e.g., lobaplatin, doxorubicin, and irinotecan) are usually water‐soluble, which poses great difficulties for TACE treatment due to their contradictory properties.^[^ ^13^ ^]^ Currently, water‐in‐oil (W/O) lipiodol emulsion loaded with a single water‐soluble chemotherapeutic drug is a typical embolic agent in TACE procedure (**Figure** **1**a). On one hand, conventional lipiodol emulsions (cLE) have an unstable water/oil interface, leading to the rapid coalescence of droplets and phase separation, which could result in lower deposition of lipiodol and burst release of water‐soluble chemotherapeutic drugs, thereby leading to lower clinical efficacy and unsatisfactory outcomes.^[^ ^15^, ^16^ ^]^ Several approaches have been attempted to improve stability of lipiodol emulsions, including the use of contrast agents for preparing the aqueous phase of chemotherapeutic drugs, as well as altering the water‐lipiodol ratio or emulsification techniques.^[^ ^17^, ^18^, ^19^ ^]^ However, these lipiodol emulsions are still unstable. Another method is to add surfactants into the emulsions to reduce interfacial tension for long‐term stability, but such emulsions directly or indirectly cause toxicity issues.^[^ ^20^, ^21^ ^]^ On the other hand, these lipiodol‐based emulsions are essentially dependent on a single chemotherapeutic drug to inhibit tumor progression, which would make it possible for tumors to acquire resistance and limit the success of antitumor treatment.^[^ ^22^, ^23^ ^]^ Some studies have shown that delivering multiple chemotherapy drugs directly through a catheter to the hepatic artery for combination therapy may help decrease tumor resistance.^[^ ^24^, ^25^ ^]^ However, due to the lack of effective chemotherapy drug carriers, these chemotherapy drugs may rapidly enter the systemic circulation, increasing the risk of systemic adverse reactions.^[^ ^26^, ^27^ ^]^ Moreover, simultaneous loading of multiple chemotherapy drugs into embolic material may result in unexpected pharmacodynamic interactions.^[^ ^28^ ^]^ Therefore, developing a stable lipiodol emulsion that can achieve sustained release of therapeutic drugs and decrease the probability of resistance through distinct antitumor mechanisms is highly desired but remains a significant challenge.

Herein, we successfully develop a novel lobaplatin‐loaded lipiodol‐based emulsion (ICN‐LPE) with a stable water/oil interface, chemotherapy, and chemodynamic therapy properties via stabilization of the lipiodol Pickering emulsion (LPE) with carefully designed iron‐doped carbon nanozymes (ICN). The stable water/oil interface of LPE and the porous structure of ICN can effectively prevent the burst release of water‐soluble chemotherapeutic drugs (i.e., lobaplatin), thereby improving the efficacy of chemotherapy. Meanwhile, ICN can catalyze the generation of excessive highly toxic reactive oxygen species (ROS) in tumor cells via a Fenton‐like reaction and consume glutathione (GSH), inducing chemodynamic therapy (Figure 1b). In vitro experiments show that ICN have significant peroxidase catalytic activity and hemocompatibility, while ICN‐LPE is capable of releasing lobaplatin in a stable manner over a 48‐h period. The New Zealand rabbit liver VX2 tumor model further confirms that ICN‐LPE can effectively inhibit tumor growth in TACE, presenting an excellent antitumor effect in vivo. Thus, ICN‐LPE shows promising clinical applicability for TACE in the treatment of liver cancer.

## 2 Results and Discussion

### 2.1 Preparation and Characterization of ICN

**Figure 2:**
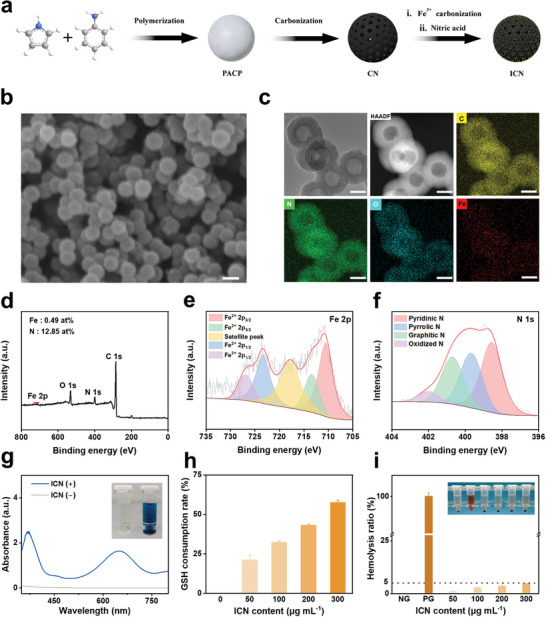
Characterization of ICN. a) Schematic illustration of the synthesis of ICN. b) SEM image of ICN (scale bar = 100 nm). c) TEM image and EDS elemental mapping (C, N, O, and Fe) of ICN (scale bars = 50 nm). d) XPS full spectrum of ICN. High‐resolution XPS spectra of e) Fe 2p and f) N 1s for ICN. g) UV–vis absorbance spectra of TMB catalyzed by ICN in the presence of H_2_O_2_. h) GSH consumption rates by ICN at various content. i) Hemolysis ratios of the red blood cells from rabbits incubated with ICN at various content. The data are presented as the mean ± standard deviation (SD) (*n* = 3). [Correction added on 12 December 2024, after first online publication: figure 2 is replaced with the updated version and the related description is revised in the Results and Discussion section.]

The synthesis process of ICN is illustrated in **Figure** **2**a. Firstly, the polyaniline‐*co*‐polypyrrole (PACP) hollow spheres were prepared and carbonized to obtain hollow carbon nanospheres. Subsequently, after secondary carbonization for the doping of iron and soaking in concentrated nitric acid, ICN were obtained. The morphology of the as‐obtained ICN was characterized by scanning electron microscopy (SEM) and transmission electron microscopy (TEM). As demonstrated in the SEM image, ICN exhibit a uniform spherical morphology (Figure 2b) with a mean diameter of 97 nm (Figure S1, Supporting Information). The TEM images reveal the hollow spherical morphology of ICN. The energy dispersive spectroscopy (EDS) of ICN demonstrates the presence and homogeneous distribution of C, N, O, and Fe elements (Figure 2c). X‐ray diffraction (XRD) analysis reveals a broad and weak graphite (002) diffraction peak, with no detected diffraction peaks from Fe nanoparticles, indicating that the Fe atoms are well dispersed in the amorphous carbon skeleton of ICN (Figure S2, Supporting Information).^[^ ^29^ ^]^ According to the nitrogen adsorption/desorption measurement, the Brunauer–Emmett–Teller (BET) surface area of ICN is calculated to be 301 m^2^ g^−1^ with well‐developed micro‐, meso‐ and macropores (Figure S3, Supporting Information), indicative of a porous structure. The chemical composition of ICN is further investigated by X‐ray photoelectron spectroscopy (XPS). The content of Fe and N elements is 0.49 at% and 12.85 at%, respectively (Figure 2d). The Fe peak can be fitted to Fe^2+^ (710.5 and 723.3 eV) and Fe^3+^ (713.3 and 726.8 eV), and the pyridinic N (398.5 eV) and pyrrolic N (399.6 eV) could serve as attachment sites for the formation of Fe–N_x_ moieties, suggesting the good coordination of Fe and N atoms (Figure 2e,f).^[^ ^30^ ^]^ Fe^2+^ is expected to catalyze the production of hydroxyl radicals (·OH) via a Fenton‐like reaction in the presence of H_2_O_2_. As expected, using 3,3′,5,5′‐tetramethylbenzidine (TMB) as a marker,^[^ ^31^ ^]^ ICN exhibit excellent peroxidase‐like activity (Figure 2g) and show content‐dependent (Figure S4, Supporting Information). To directly verify the generation of ·OH, a typical component of ROS, dimethylpyridine nitrogen oxide (DMPO) was used as a spin trap to capture DMPO‐OH adducts for electron spin resonance (ESR) detection.^[^ ^32^ ^]^ In the presence of ICN and H_2_O_2_, a strong characteristic signal ratio of 1:2:2:1 is detected, confirming the Fe‐triggered generation of ·OH (Figure S5, Supporting Information). Excessive ROS in cells may induce tumor cell ferroptosis.^[^ ^33^ ^]^ In addition, glutathione peroxidase 4 (GPX4) is involved in the detoxification of ROS by employing GSH as a cofactor, and the consumption of GSH holds the potential to inactivate GPX4 and enhance ROS accumulation.^[^ ^34^, ^35^ ^]^ To investigate the ability of ICN to consume GSH, in vitro simulation experiments were conducted. The amount of remaining GSH significantly decreases with the increase of ICN content, and when the GSH concentration is 1 mg mL^−1^ and the ICN content is 300 µg mL^−1^, the GSH consumption rate is ≈57% (Figure 2h). This result indicates that ICN have the ability to consume GSH in a content‐dependent manner. These results show the potential chemodynamic therapy of ICN to induce tumor cell ferroptosis via the excessive accumulation of ROS caused by both the enhanced production of ROS and consumption of intracellular GSH, thereby potentially overcoming chemoresistance.^[^ ^36^, ^37^, ^38^ ^]^

Given that ICN injected into tumor‐supplying arteries via a microcatheter are directly contacted with blood, the potential to induce erythrocyte hemolysis is a concern.^[^ ^39^ ^]^ Thus, the hemocompatibility of ICN was evaluated by hemolysis experiments. ICN suspensions at various content (50, 100, 200, and 300 µg mL^−1^) were incubated with rabbit erythrocytes at 37 °C for 4 h. Optical image reveals that the solution of red blood cells incubated with ICN has a clear or slightly yellow appearance, which is similar to that of the negative control group (Figure 2i). Quantitative analysis shows that the hemolysis rate increases from 1% to 4.7% with the increase of the ICN content (Figure 2i). Even at 300 µg mL^−1^, the hemolysis rate remains below the safety threshold of 5%, suggesting excellent hemocompatibility of ICN. To comprehensively evaluate the biosafety of ICN, a subcutaneous implantation experiment was conducted in C57BL/6 mice. PBS and ICN suspension were injected into subcutaneous pockets on the dorsal surface of the mice to assess potential subcutaneous inflammatory responses. Encouragingly, no abnormal behavior is noted within the given time interval, and no apparent damage to the skin or surrounding soft tissue is observed in the ICN‐injected regions. Mice were subsequently euthanized at day 14 after injection. Hematoxylin eosin (HE) staining reveals a normal appearance of the subcutaneous muscle layers and skin tissue, with no significant signs of inflammation. In addition, an immunostaining technique was utilized to assess the expression of IL‐6 (an inflammatory factor). No significant increase in the expression level of IL‐6 is observed in the ICN‐injected regions (Figure S6, Supporting Information), further confirming the good biocompatibility of ICN.

### 2.2 Preparation and Characterization of ICN‐LPE

**Figure 3:**
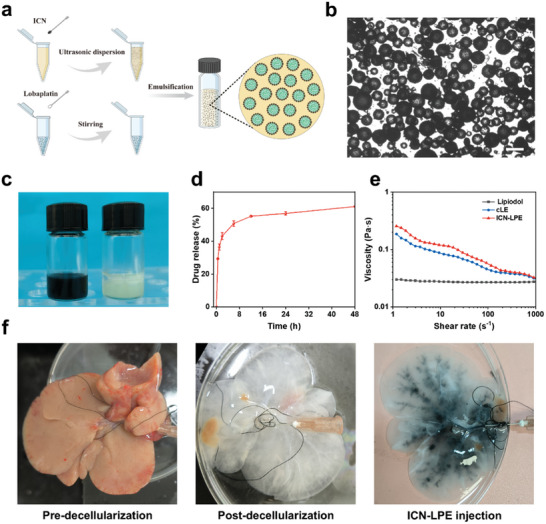
Characterization of ICN‐LPE. a) Schematic representation of the process for ICN‐LPE preparation and corresponding emulsification mechanism. b) Optical microscopic image of ICN‐LPE (scale bar = 200 µm). c) Digital photo of ICN‐LPE and cLE after standing for 6 h. ICN‐LPE displays stability and cLE shows phase separation. d) Profile of lobaplatin released from ICN‐LPE. The data are presented as the mean ± SD (*n* = 3). e) Viscosity vs shear rate of lipiodol, cLE, and ICN‐LPE. f) Digital photos of decellularized liver tissue and distribution of ICN‐LPE in hepatic arteries.

As inorganic nanomaterials, ICN could also be used to prepare stable Pickering emulsions.^[^ ^40^, ^41^ ^]^ As illustrated in **Figure** **3**a, ICN were dispersed in lipiodol to obtain the mixture of ICN and lipiodol (ICN/L). Then, lobaplatin aqueous solution was subsequently added into ICN/L at a water‐lipiodol ratio of 1:3, which is commonly used in clinical applications.^[^ ^42^ ^]^ Stable W/O ICN‐LPE was prepared by the shear action of a high‐speed stirrer.^[^ ^43^, ^44^, ^45^ ^]^ As expected, the droplet diameter decreases progressively with the increase of ICN content and stabilizes at ≈127 µm at an ICN content of 10 mg mL^−1^, which is chosen for preparing ICN‐LPE (Figure S7, Supporting Information). As shown in Figure 3b, dispersed water droplets are clearly visible within the continuous lipiodol phase in ICN‐LPE by optical microscopy, and ICN could be differentiated at the water/lipiodol interface. In contrast to the rapid phase separation in cLE, ICN‐LPE remains stable after 6 h (Figure 3c). The release rate of chemotherapeutic drugs from emulsion is closely related to the emulsion stability. To explore the sustained drug release functionality of ICN‐LPE, a simulation experiment in vitro was conducted. After incubation in PBS, the cumulative release rates of lobaplatin are 29%, 43%, 50%, 56%, and 61% at 0.5, 2, 6, 24, and 48 h, respectively (Figure 3d). This result suggests that ICN‐LPE could reduce the burst release of lobaplatin, which could be attributed to the stable water/lipiodol interface for preventing phase separation and the porous structure of ICN for certain adsorption of lobaplatin.

Another key characteristic of lipiodol emulsion is viscosity, which directly affects its injectability through a microcatheter and its ability to homogenously distribute in the hepatic artery.^[^ ^46^, ^47^ ^]^ We tested the viscosities of lipiodol, cLE, and ICN‐LPE using a rheometer. Similar to cLE, ICN‐LPE shows typical shear‐thinning characteristics. As the shear rate increases, the viscosity of ICN‐LPE decreases to a level equivalent to lipiodol, indicating that ICN‐LPE has excellent injectability for TACE (Figure 3e). Subsequently, a decellularization technique was used to make Sprague Dawley rat liver tissue transparent to clearly display vascular structure. As shown in Figure 3f, ICN‐LPE is well deposited within the arteries after being injected into the hepatic arteries. These findings strongly support further exploration of the application of ICN‐LPE for TACE in vivo.

### 2.3 Synergistic Effect of ICN and Lobaplatin on HepG2 Cells

**Figure 4:**
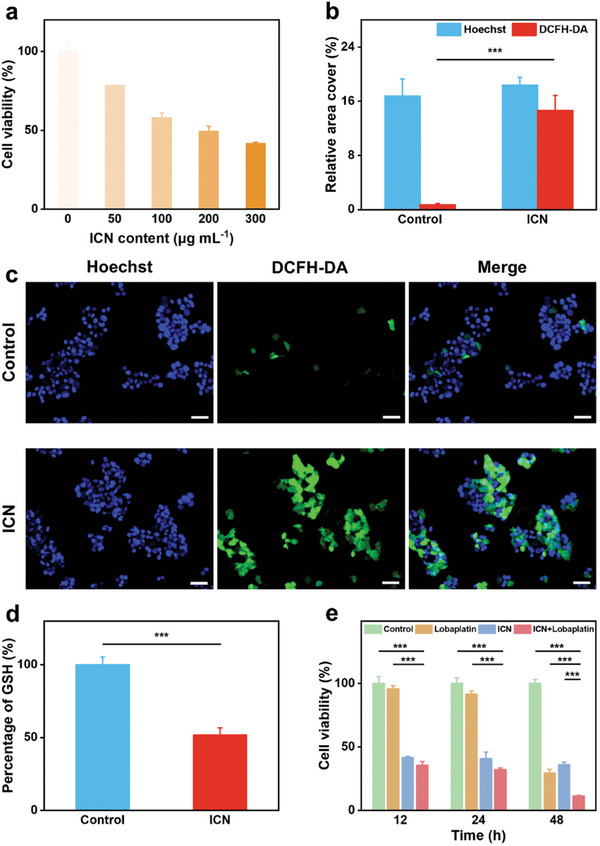
Antitumor efficacy and mechanism of ICN synergizing with lobaplatin. a) Viability rates of HepG2 cells incubated with ICN at various content for 12 h. The data are presented as the mean ± SD (*n* = 3). b) Comparison of the fluorescence ratio of Hoechst and ROS of the control and ICN groups. The data are presented as the mean ± SD (*n* = 3).c) Fluorescence images of ROS generation in HepG2 cells stained with DCFH‐DA (scale bars = 50 µm). d) GSH levels in HepG2 cells of the control and ICN groups. The data are presented as the mean ± SD (*n* = 5). e) Viability rates of HepG2 cells of the control, lobaplatin, ICN, and ICN + lobaplatin groups after incubation for 12, 24, and 48 h.

Firstly, to evaluate the antitumor efficacy of ICN in vitro, CCK‐8 assay was used to quantitatively assess the viability rate of HepG2 cells. With the increase of ICN content, the viability rate of HepG2 cells decreases significantly, indicating that ICN inhibits the proliferation of HepG2 cells in a content‐dependent manner. At an ICN content of 300 µg mL^−1^, the cell viability rate is low to 41.4% at 12 h (**Figure** **4**a). To dissect the underlying mechanism of ICN inducing cell death, 2′,7′‐dichlorofluorescein diacetate (DCFH‐DA) was used to evaluate intracellular ROS level. As shown in Figure 4b,c, significant green fluorescence is observed in ICN‐treated HepG2 cells, and statistically, the fluorescence intensity of the ICN group is over 4 times higher than that of the control group. The inhibitory effect of ICN on HepG2 cells proliferation could be attributed to oxidative damage caused by ROS. Moreover, the intracellular GSH level of the ICN group decreases to 51% compared with the control group (Figure 4d). These results mean that ICN can effectively increase intracellular ROS level by catalytical production of ROS and consumption of intracellular GSH. Excessive ROS accumulation will disrupt the intrinsic equilibrium within the cellular microenvironment, associated with mitochondrial dysfunction.^[^ ^48^ ^]^ JC‐1 probe was used to investigate changes in the mitochondrial membrane potential of HepG2 cells. Compared with the control group, the cells incubated with ICN present noticeable decreases in red fluorescence accompanied with the appearance of green fluorescence (Figure S8, Supporting Information), suggesting that ROS‐related oxidative stress triggered by ICN can reduce the mitochondrial membrane potential and induce HepG2 cell death through impairing the mitochondrial respiratory chain.^[^ ^35^ ^]^

As a cytotoxic drug, lobaplatin can inhibit the proliferation of cancer cells by preventing DNA replication and transcription, and commonly used in chemotherapy, especially in TACE for the treatment of hepatic carcinoma and liver metastases of colorectal cancer.^[^ ^49^, ^50^, ^51^ ^]^ Then, the synergistic antitumor effect of the combination of ICN and lobaplatin was assessed. The HepG2 cell viability rates of the lobaplatin (5 µg mL^−1^), ICN (300 µg mL^−1^), and ICN + lobaplatin groups were measured at 12, 24, and 48 h. Interestingly, we find that the cell viability rate of the ICN group decreases mainly at 12 h, and the cell viability rate of the lobaplatin group decreases at 48 h, which is consistent with their antitumor mechanisms that ICN can work upon contact with tumor cells, while lobaplatin mainly inhibits cell proliferation in a relatively later stage. Compared with ICN and lobaplatin, ICN + lobaplatin continuously inhabits HepG2 cell proliferation throughout the experimental period and exhibits a significant synergistic antitumor effect through cytotoxicity and induction of ferroptosis (Figure 4e), which is expected to reduce chemoresistance.^[^ ^52^ ^]^

### 2.4 In vivo Therapeutic Efficacy of ICN‐LPE on VX2 Tumors

**Figure 5:**
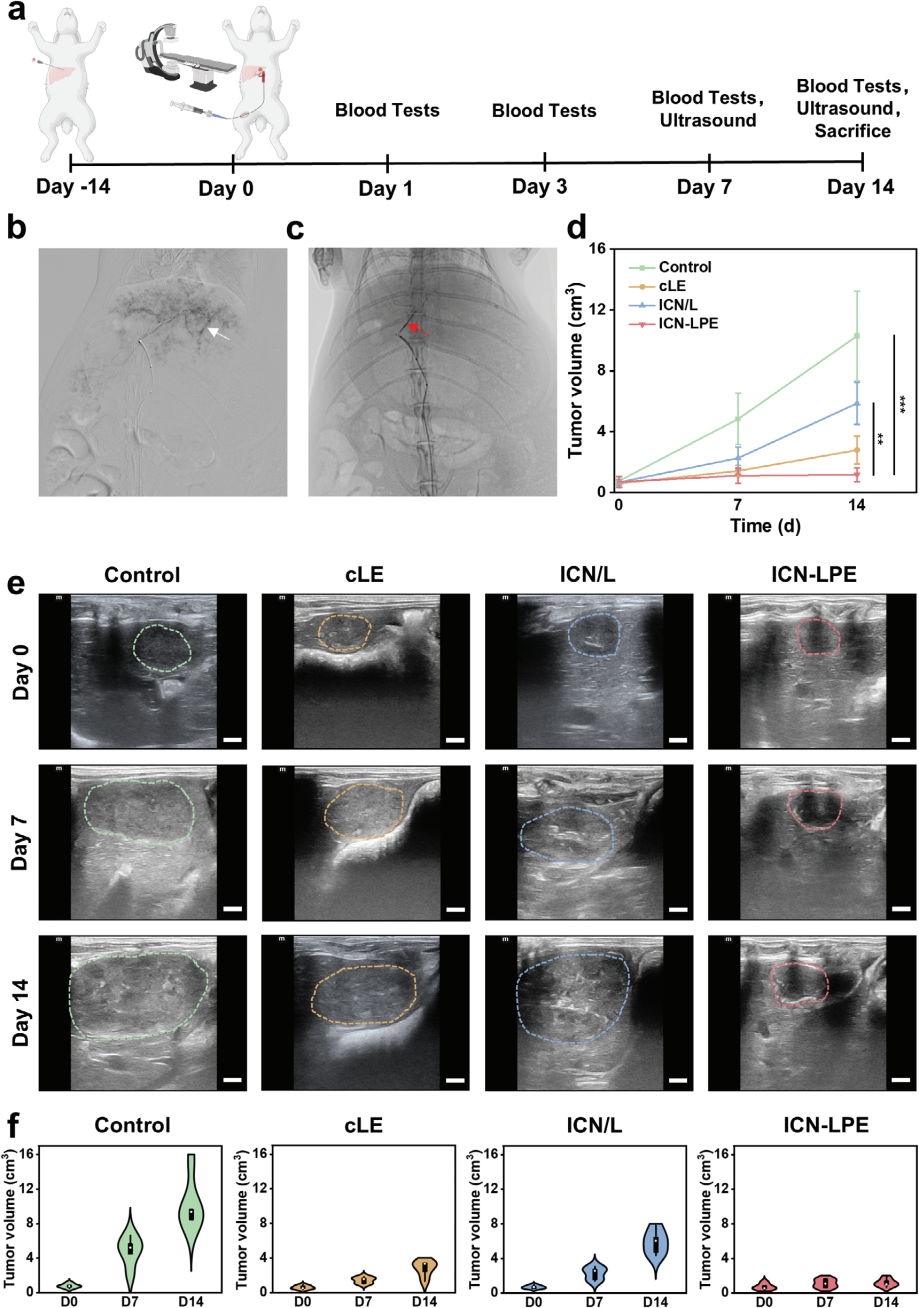
Embolization and antitumor effect of ICN‐LPE in VX2 tumor models. a) Schematic diagram of the experimental schedule. b) DSA reveals “tumor staining”, which is a characteristic of the tumor with an abundant blood supply. c) ICN‐LPE is injected into the tumor tissue through the tumor‐supplying artery. d) Tumor growth curves of different groups. e) Ultrasound images of tumors in the control, cLE, ICN/L, and ICN‐LPE groups at days 0, 7, and 14 (scale bars = 500 µm). f) Tumor growth indices (violin plots) for individual groups. The data are presented as the mean ± SD (*n* = 5).

Encouraged by the results in vitro, liver VX2 tumors in rabbits were established to assess the therapeutic efficacy of ICN‐LPE in vivo. As shown in **Figure** **5**a, 20 New Zealand rabbits with VX2 tumors in the left liver lobes were divided into four groups: 1) control, 2) cLE, 3) ICN/L, and 4) ICN‐LPE. A 4F vascular sheath was inserted into the right femoral artery (Figure S9, Supporting Information), and a 2.3F microcatheter was then selectively inserted into the left hepatic artery. Digital subtraction angiography (DSA) clearly reveals the rich microvasculature surrounding and within the VX2 tumors (Figure 5b). PBS, cLE, ICN/L, or ICN‐LPE was injected into the left hepatic artery via a microcatheter (Figure 5c), and tumor sizes were monitored by ultrasound and recorded (Figure 5d,e). The tumors volume in the control group grow rapidly from 0.7 ± 0.2 to 10.0 ± 2.9 cm^3^ within 14 days. Conversely, the tumors volume in the ICN‐LPE group is only 1.2 ± 0.5 cm^3^ at day 14, which is obviously less than those in the cLE (2.8 ± 0.9 cm^3^) and ICN/L (5.9 ± 1.4 cm^3^) groups (Figure 5f), well consistent with the in vitro cellular experiment results.

**Figure 6:**
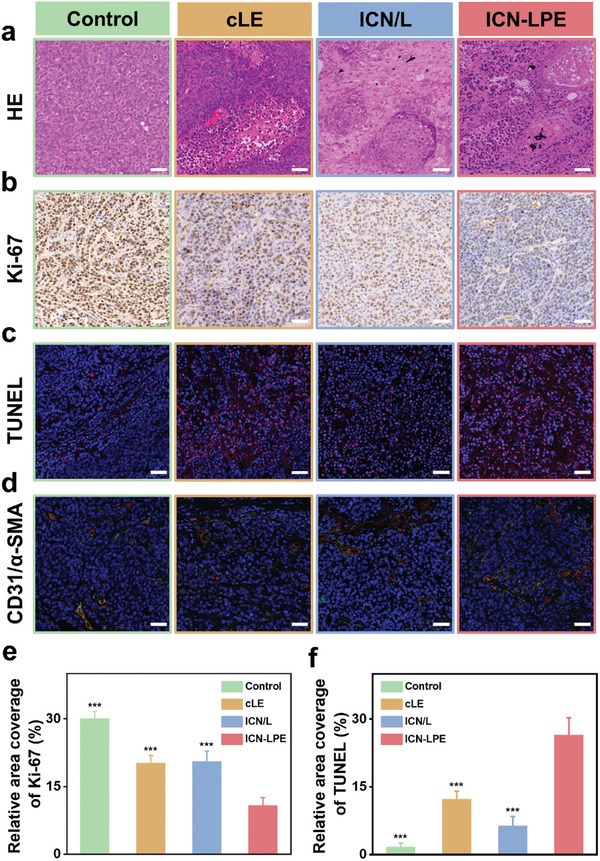
Histological analysis of VX2 tumors at day14 after TACE. a–d) HE staining (a), Ki‐67 immunohistochemical staining (b), and TUNEL (c) and CD31/α‐SMA (d) immunofluorescence images (scale bars = 50 µm). e–f) Quantitative analysis of Ki‐67 (e) and TUNEL (f) in each group. The data are presented as the mean ± SD (*n* = 5).

HE staining reveals that ICN are deposited within the tumor tissues of the ICN/L and ICN‐LPE groups and tumor cell nuclei show shrinkage and fragmentation in the ICN‐LPE group (**Figure** **6**a). Furthermore, among the four groups, the expression level of Ki‐67 is the lowest (Figures 6b and 6e), and the expression level of terminal deoxynucleotidyl transferase dUTP nick end labeling (TUNEL) is the highest in the ICN‐LPE group (Figures 6c and 6f), indicating that ICN‐LPE could lead to significant inhibition of cell proliferation and induction of tumor cell death. Considering that embolization therapy might induce neovascularization in tumor tissues, which is not conducive to successful cancer treatment,^[^ ^53^, ^54^ ^]^ we assessed the formation of new capillaries via double immunofluorescence staining of CD31 and α‐SMA. The results reveal no significant increase in CD31 or α‐SMA expression in the ICN‐LPE group compared with the cLE and ICN/L groups, further suggesting the therapeutic effectiveness of ICN‐LPE (Figure 6d).

To assess the safety of ICN‐LPE for TACE, biochemical and hematological parameters were simultaneously detected during the animal experiments. Usually, vascular embolization could not only cause damage to liver cancer cells, but also to hepatocytes in some extent.^[^ ^55^ ^]^ After TACE for 7 days, significant increases in alanine aminotransferase (ALT) levels of the lipiodol‐containing groups (especially the ICN‐LPE group) are observed, suggesting a short‐term hepatocyte damage. Reassuringly, the ALT levels of all groups have completely returned to within the normal range at day 14 (Figure S10a, Supporting Information). In terms of renal function, there is no significant difference in blood creatinine levels among all rabbits throughout the experimental period (Figure S10b, Supporting Information). Hematological analysis reveals the total white blood cell counts in the ICN‐LPE, cLE, and ICN/L groups increase at day 1 and day 3 after TACE, which is likely related to surgical stress after embolization of the artery, and restore to normal level at day 7 after TACE (Figure S10c, Supporting Information). Additionally, pathological analysis reveals that no significant abnormal structural changes on the major organs (heart, lung, kidney, and spleen) are observed in the ICN‐LPE group (Figure S11, Supporting Information). These results demonstrate that ICN‐LPE exhibits a good safety performance in TACE.

## 3 Conclusion

In conclusion, we have developed a novel lobaplatin‐loaded lipiodol Pickering emulsion, which integrates conventional chemotherapy with chemodynamic therapy, thereby markedly enhancing antitumor efficacy in TACE. ICN‐LPE not only facilitates the sustained release of chemotherapeutic drugs but also induces excessive accumulation of highly toxic ROS within tumor cells through the production of ROS and the consumption of GSH. These synergistic multimodal cancer therapies can result in significant growth inhibition of liver VX2 tumors in New Zealand rabbits. Our work may offer a novel direction for the development of embolic materials for TACE and highlight the immense potential for future application in tumor interventional therapy.

## 4 Experimental Section

The Experimental Section is available in the Supporting Information.

## Conflict of Interest

The authors declare no conflict of interest.

## Supporting information



Supporting Information

## Data Availability

The data that support the findings of this study are available from the corresponding author upon reasonable request.
