# Supplementary material for: A Lipiodol Pickering Emulsion Stabilized by Iron‐Doped Carbon Nanozymes for Liver Transarterial Chemoembolization
Source: Adv Sci (Weinh). 2024 Dec 10;12(5):2410873. doi: 10.1002/advs.202410873 (PMC11791992; doi:10.1002/advs.202410873)
Supplement: Supplementary file 1 — Supporting Information [file ADVS-12-2410873-s001.docx]

A Lipiodol Pickering Emulsion Stabilized by Iron-Doped Carbon Nanozymes for Liver Transarterial Chemoembolization

Xiancheng Xia, Yang Li, Rongkang Huang, Yuanbin Wang, Wenxuan Xiong, Hui Zhou, Min Li*, Xidong Lin*, Youchen Tang*, Bo Zhang*

**X. Xia, B. Zhang**

Department of Interventional Center

Biomedical Innovation Center

The Sixth Affiliated Hospital, Sun Yat-Sen University

Guangzhou 510655, P. R. China

E-mail: zhangb28@mail.sysu.edu.cn

Y. Li, Y. Tang

The Eighth Affiliated Hospital, Sun Yat-sen University

Shenzhen 518033, P. R. China

E-mail: tangych7@ mail.sysu.edu.cn

**R. Huang, Y. Wang, W. Xiong**

Department of General Surgery (Colorectal Surgery)

Guangdong Provincial Key Laboratory of Colorectal and Pelvic Floor Diseases

Biomedical Innovation Center

The Sixth Affiliated Hospital, Sun Yat-Sen University

Guangzhou 510655, P. R. China

H. Zhou

PCFM Lab, School of Chemistry, Sun Yat-sen University

Guangzhou 510006, P. R. China

M. Li

Department of Gastrointestinal Surgery

The Affiliated Dongguan Songshan Lake Central Hospital, Guangdong Medical University

Dongguan 523326, P. R China

E-mail: drlimin@dingtalk.com

X. Lin

Future Technology School, Shenzhen Technology University

Shenzhen 518118, P. R China

E-mail: linxidong@sztu.edu.cn

**Materials**

Triton X-100, potassium persulphate (KPS), aniline, pyrrole, 1,10-phenanthroline, Fe (III) nitrate nonahydrate, and dimethyl sulfoxide (DMSO) were purchased from Shanghai Macklin Biochemical Co., Ltd. (China). Lipiodol was acquired from Jiangsu Hengrui Pharmaceutical Co., Ltd. (China). Lobaplatin was acquired from Hainan Changan International Pharmaceutical Co., Ltd. (China). Cell Counting Kit-8 (CCK-8) and phosphate buffer saline (PBS, 0.01 M, pH 7.4) were purchased from Dalian Meilun Biotechnology Co., Ltd. (China). HepG2 cells were provided by the Procell Life Science & Technology Co., Ltd. (China). Fetal bovine serum (FBS) and minimum essential medium (MEM) were purchased from Gibco-BRL Life Technologies, Inc. (USA). Glutathione Content Assay Kit was purchased from Beijing Solarbio Science & Technology Co., Ltd. (China). Reactive Oxygen Species Assay Kit and Mitochondrial Membrane Potential Assay Kit with JC-1 were purchased from Beyotime Biotech Inc. (China). Male Sprague Dawley (SD) rat and male C57BL/J mice were provided by Laboratory Animal Center of Sun Yat-Sen University (China). New Zealand rabbits were purchased from Huateng BioScience Co., Ltd. (China).

**Synthesis of ICN**

In a typical synthesis, 2.28 mL of aniline, 1.75 mL of pyrrole, and 0.34 mL of Triton X-100 were initially dissolved in 360 mL of deionized (DI) water. The suspension was stirred for 30 min followed by 30 min sonication, and precooled in ice bath. Then, KPS solution was added into the precooled suspension and allowed to polymerize for 16 h at 0 °C. After polymerization, the product was washed with DI water until the filtrate became colorless and freeze-dried, obtaining polyaniline-*co*-polypyrrole (PACP). Subsequently, the as-prepared PACP was carbonized at 900 °C for 10 h under a nitrogen atmosphere with a heating rate of 5 °C min^−1^ to obtain carbon nanospheres.^[1]^ Next, 200 mg of Fe (III) nitrate nonahydrate was dissolved in 2 mL of a water-ethanol mixture (1:9 v/v) and subsequently added to 2 mL of water-ethanol solution containing 1,10-phenanthroline (300 mg) and carbon nanospheres (400 mg). The resultant mixture was stirred at 60 °C for 3 h, followed by drying at 80 °C for 12 h. The resulting black powder was then subjected to thermal treatment at 600 °C for 2 h under a nitrogen atmosphere. Afterward, the product was treated with 2 M hydrochloric acid at 80 °C for 2 h and collected after washing and freeze-drying. Finally, the product was soaked in concentrated nitric acid for 16 h, followed by washing and freeze-drying, yielding target sample ICN.

**Preparation of cLE, ICN/L, and** **ICN-LPE**

The cLE was prepared by emulsifying lipiodol with an aqueous solution containing lobaplatin (5 mg mL^−1^) under high-speed stirring (5,000 rpm for 1 min). To prepare ICN/L, ICN (10 mg mL^−1^) were mixed with lipiodol under ultrasonic dispersion for 10 min. Then, the performed ICN/L was emulsified with an aqueous solution containing lobaplatin (5 mg mL^−1^) under high-speed stirring (5,000 rpm for 1 min), resulting in ICN-LPE. Both cLE and ICN-LPE were prepared at a water-lipiodol ratio of 1: 3. The viscosities of lipiodol, cLE, and ICN-LPE were measured with a rotational rheometer (Kinexus pro+, Malvern, UK).

**Material characterization**

Scanning electron microscopy (SEM, Sigma 300, Zeiss, Germany) was used to examine the structure of ICN. The morphologies and corresponding energy dispersive X-ray spectroscopy (EDS) spectra of ICN were acquired from a transmission electron microscope (TEM, TalosF200S G2, Thermo Fisher Scientific, USA). X-ray diffraction (XRD) pattern was recorded on a diffractometer (D MAX 2200 VPC, Rigaku, Japan) using Cu Kα radiation with a 2θ ranges from 10-80°. X-ray photoelectron spectroscopy spectra were collected on a scanning X-ray microprobe (XPS, K-Alpha, Thermo Fisher Scientific, USA). The N_2_ adsorption-desorption isothermal curve and corresponding pore-size distribution were measured by Accelerated Surface Area and Porosimetry System (ASAP 2460, Micromeritics, USA). The peroxidase-like activity of ICN was assessed by using TMB as the substrate in the presence of H_2_O_2_. The absorbance of TMB solution diluted 10 times was recorded through spectrophotometer (UV2010, Hitachi, Japan) at various ICN content in the presence of H_2_O_2_. The electron spin resonance (ESR) spectra were characterized by an ESR spectrometer (JES-FA200, JEOL, Japan).

**In vitro GSH consumption assay**

ICN were mixed at various content (50, 100, 200, and 300 μg mL^−1^) with 3 mL of GSH solution (1 mg mL^−1^), and the suspensions were incubated at 37 °C for 6 h, centrifuged at 8,000 rpm for 5 min. Subsequently, 90 μL supernatants were added to the wells of a 96-well plate, and 40 μL of DTNB-DMSO solution (1.5 mg mL^−1^) was also added to each well. Their absorbances at 412 nm were recorded by a microplate reader (Multiskan SkyHigh, Thermo Fisher Scientific, USA) to evaluate the amount of remaining GSH.

**Hemolysis assay**

The hemolysis assay was used to evaluate the biosafety of ICN. Rabbit red blood cells were diluted with H_2_O, PBS, and ICN suspensions (50, 100, 200, and 300 μg mL^−1^), and the mixtures were incubated at 37 °C for 4 h, centrifuged at 3,000 rpm for 10 min. The obtained supernatants were collected, and their absorbances at 540 nm were recorded by a microplate reader. Red blood cells treated with H_2_O were used as positive control, and treated with PBS were used as negative control. Hemolysis rate (%) = (OD_sample_ − OD_-_) / (OD_+_ − OD_-_) × 100%. Where the OD_sample_, OD_+_, and OD_-_ represent the absorbance at 540 nm of the sample, positive control, and negative control groups, respectively.

**In vivo biocompatibility assay**

All animal-related experimental procedures in this study were approved by the guidelines of the Animal Ethics Committee for Huateng BioScience Co., Ltd. (China, license number: C202312-1). Biocompatibility was assessed by subcutaneous implantation into the dorsal skin of C57BL/6 mice. Three C57BL/6 mice were anesthetized using isoflurane (1%~2% isoflurane in oxygen). 100 μL ICN suspension (2.5 mg mL^−1^) and 100 μL PBS were injected subcutaneously into distinct regions. The mice were then observed for physical or behavioral changes. All the mice were euthanized at day 14 after implantation, and skin samples were collected and fixed with 4% paraformaldehyde for 24 h for subsequent HE staining and immunohistochemical staining for the inflammatory factor IL-6.

**In vitro sustained release of lobaplatin assay**

ICN-LPE (2 mL) was added to dialysis bag and then immersed in PBS solution (50 mL) at pH 7.4. The entire experimental system was placed on a shaker at 37 °C. At scheduled time (0.5, 1, 2, 6, 12, 24, and 48 h), 3 mL of the solution was taken out (replaced by 3 mL of fresh PBS) to detect the lobaplatin concentration. The concentration of lobaplatin was quantified by measuring the absorbance at 210 nm using a UV spectrophotometer.

**Liver decellularization process**

The SD rat was anesthetized with isoflurane (1%~2% isoflurane in oxygen). A midline incision was made in the upper abdomen to open the peritoneal cavity, exposing the portal vein. The portal vein was punctured with an indwelling needle, and 2 mL of heparin (100 U mL^−1^) was injected as an anticoagulant. The indwelling needle was secured in the portal vein, and the liver was removed intact. The liver was stored at –80 °C for 24 h and then thawed at room temperature. The liver was perfused with PBS for 1 h, 1% Triton X-100 for 3 h, 0.1% sodium dodecyl sulfate for 3 h, and PBS for 1 h successively. The perfusion rate was 3 mL min^−1^.

**In vitro antitumor effect assay**

HepG2 cells were seeded on a 96-well plate with 100 µL of MEM solution, which was supplemented with 10% FBS and 1% penicillin-streptomycin. The cells were incubated at 37 °C with 5% CO_2_. After incubation for 24 h, complete culture medium containing ICN, lobaplatin, or ICN + lobaplatin was introduced into each well to replace the initial culture medium. Following incubation periods of 12, 24, or 48 h, the culture medium was carefully removed and washed by PBS for three times. The cells were incubated with 10% CCK-8 assay under dark conditions for 1 h and OD value was recorded by the microplate reader at 450 nm absorbance to evaluate cell viability.

**Intracellular ROS assay and mitochondrial membrane potential assay**

HepG2 cells were seeded on a 48-well plate with 300 µL of MEM solution, which was supplemented with 10% FBS and 1% penicillin-streptomycin. The cells were incubated at 37 °C with 5% CO_2_. After incubation for 24 h, complete culture medium containing ICN (100 μg mL^−1^) or fresh complete culture medium was introduced into each well to replace the initial culture medium. After 6 h of incubation, intracellular ROS production and mitochondrial membrane potential of each group were assessed using the Reactive Oxygen Species Assay Kit and Mitochondrial Membrane Potential Assay Kit with JC-1, according to the manufacturer’s instructions, respectively.

**Intracellular GSH consumption assay**

HepG2 cells were seeded on a 6-well plate with 2.5 mL of MEM solution, which was supplemented with 10% FBS and 1% penicillin-streptomycin. The cells were incubated at 37 °C with 5% CO_2_. After incubation for 24 h, complete culture medium containing ICN (100 μg mL^−1^) or fresh complete culture medium was introduced into each well to replace the initial culture medium. After 6 h of incubation, intracellular GSH of each group was assessed using Glutathione Content Assay Kit, according to the manufacturer’s instructions.

**Transarterial chemoembolization in vivo**

To establish the transplanted liver tumor model, VX2 tumor tissue was cut into fragments of approximately 1 mm^3^, which were placed into a 17G puncture needle. The rabbits were fasted for 8 h and then anesthetized with Tiletamine and Zolazepam. Each rabbit was placed in the supine position and fixed on the operating table. A 2–3 cm longitudinal incision was made along the abdominal midline, and the abdominal wall was opened layer by layer to expose the left lobe of liver. The tumor tissue was inserted via 17G puncture needle into the parenchyma of the left hepatic lobe at a depth of 1 cm, and the puncture tract was sealed with tissue glue. Then, the muscles and skin were sutured layer by layer, and the wound was disinfected. To prevent postoperative infection, intramuscular injections of penicillin (100,000 U) were administered for 3 consecutive days.

Two weeks after the VX2 tumors were transplanted into left liver lobes of New Zealand rabbits, the rabbits were anesthetized and fixed on the DSA bed. A 2 cm longitudinal incision was made along the direction of femoral artery to expose the femoral artery. A 21G puncture needle was used to puncture the femoral artery, and a 0.014-inch guidewire was introduced and exchanged for a 4F vascular sheath. A 2.3F microcatheter was selectively inserted into the left hepatic artery under DSA guidance, and the tumor blood supply was confirmed by angiography. 0.3 mL of the certain emulsion was injected through the microcatheter. Finally, the femoral artery was ligated at both ends, the muscles and skin were sutured layer by layer. Intramuscular injections of penicillin (100,000 U) were administered for 3 consecutive days. To further assess the antitumor effect, tumor sizes were monitored via ultrasound at days 0, 7, and 14, and the tumor volumes were calculated as follows:

Volume = Length × Width^2^/2.

All rabbits were humanely euthanized at day 14 after TACE, following the Animal Welfare Act guidelines.

**Histological staining**

The tumor tissues were fixed in formalin for 24h, subsequently sent to Wuhan Servicebio Technology Co., Ltd. (China) for HE, Ki-67, TUNEL, and CD31/α-SMA staining. In addition, tissues from the hearts, lungs, kidneys, and spleens were also collected for HE staining.

**Biochemical analysis**

Blood samples were collected from the marginal ear artery of the experimental rabbit at days 0 1, 3, 7, and 14 after TACE, subsequently sent to Wuhan Servicebio Technology Co., Ltd. (China) to analyze hematological parameters, including alanine aminotransferase (ALT), creatinine, and white blood cell counts.

**Statistical analysis**

All statistical analysis was carried out using Origin software (Origin Lab Incorporation, Northampton, USA). Data were expressed as mean ± standard deviation (SD). Statistical differences between groups were determined by one-way analysis of variance (ANOVA). Statistical significance was marked with * *p* < 0.05, ** *p* < 0.01, and *** *p* < 0.001.


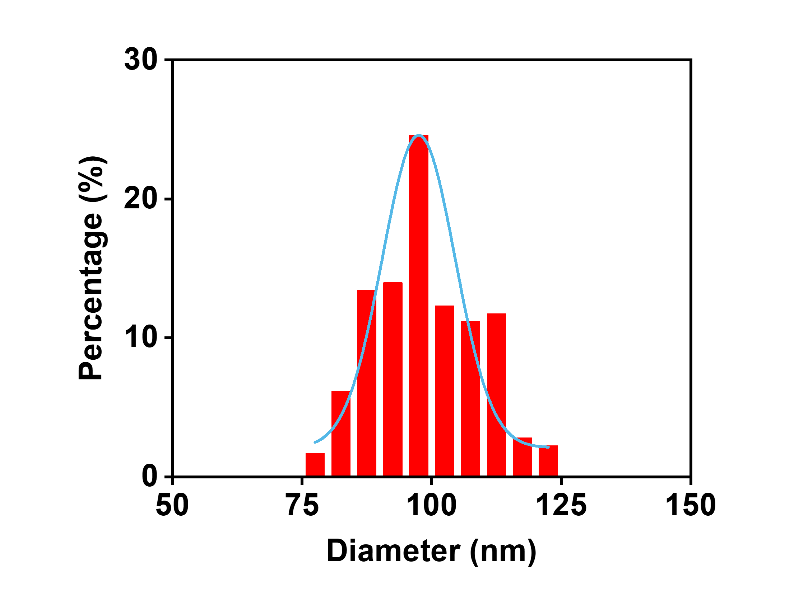


**Figure S1.** Size distribution of ICN.


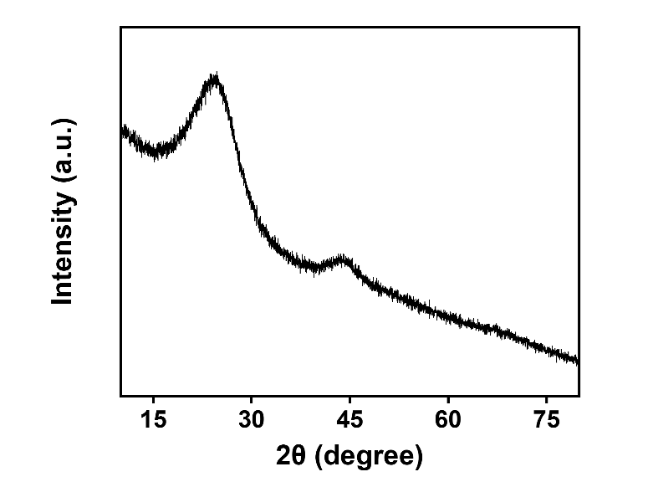


**Figure S2.** XRD spectrum of ICN.


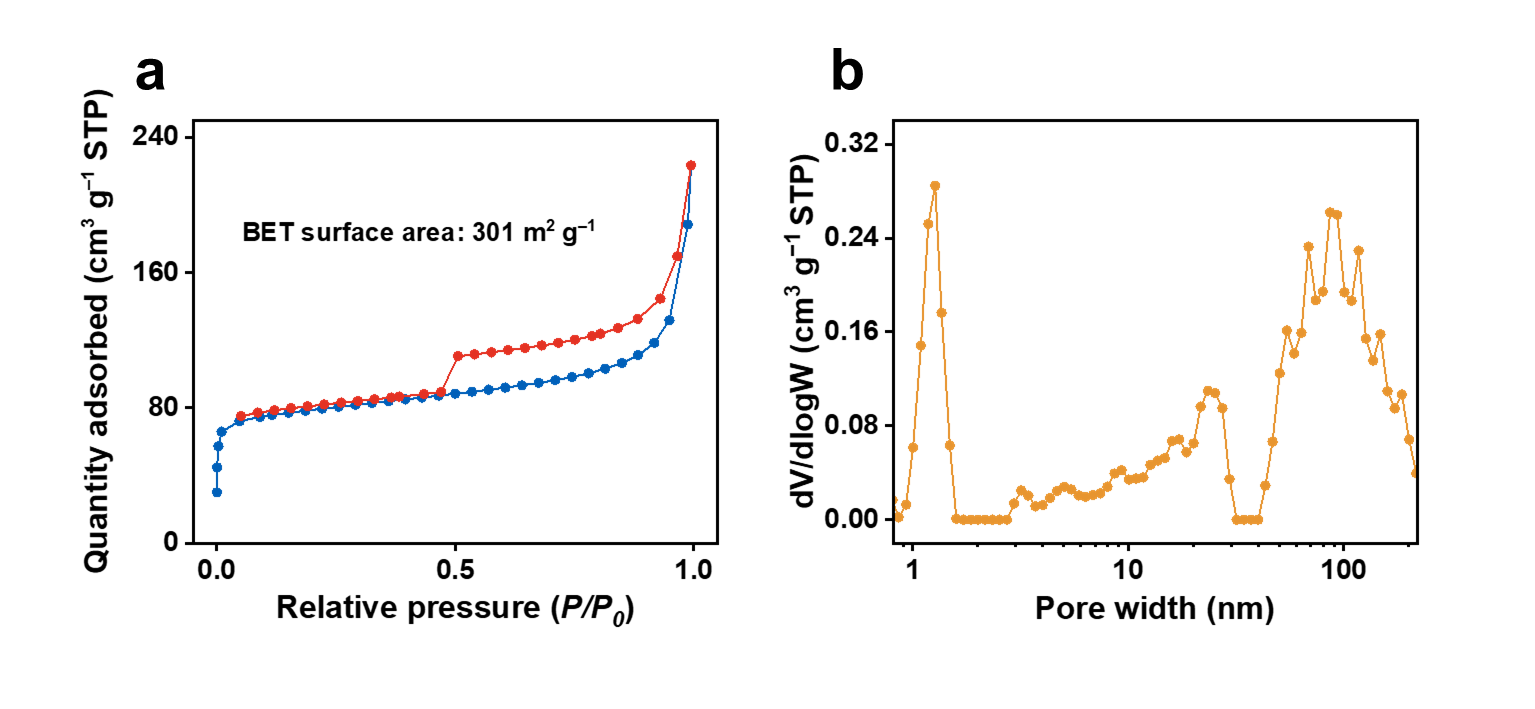


**Figure S3.** a) N_2_ adsorption–desorption isotherm of ICN and b) density functional theory pore size distribution curve of ICN.


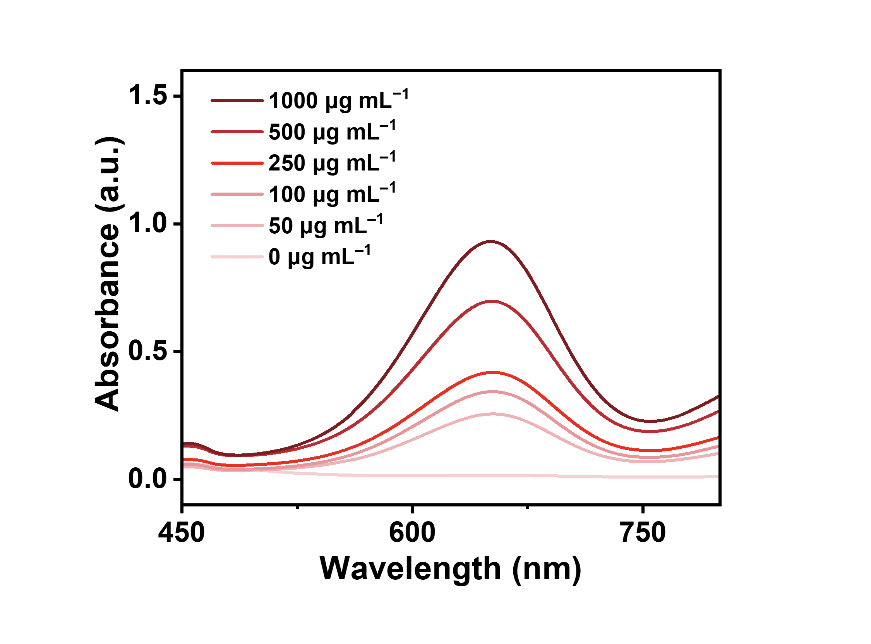


**Figure S4.** UV–vis absorbance spectra of TMB catalyzed by ICN at various content in the presence of H_2_O_2_.


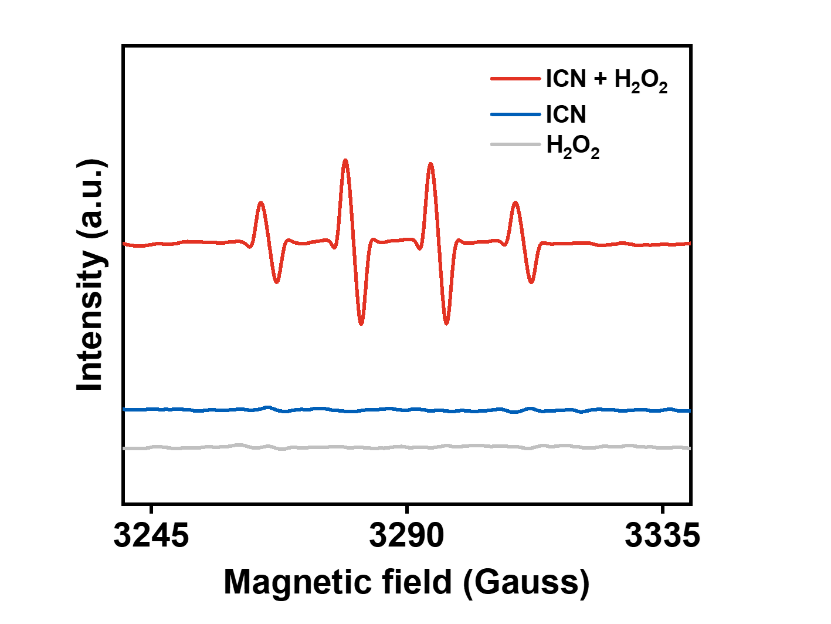


**Figure S****5.** ESR detection of ICN with DMPO as •OH trapper in the presence of H_2_O_2_.


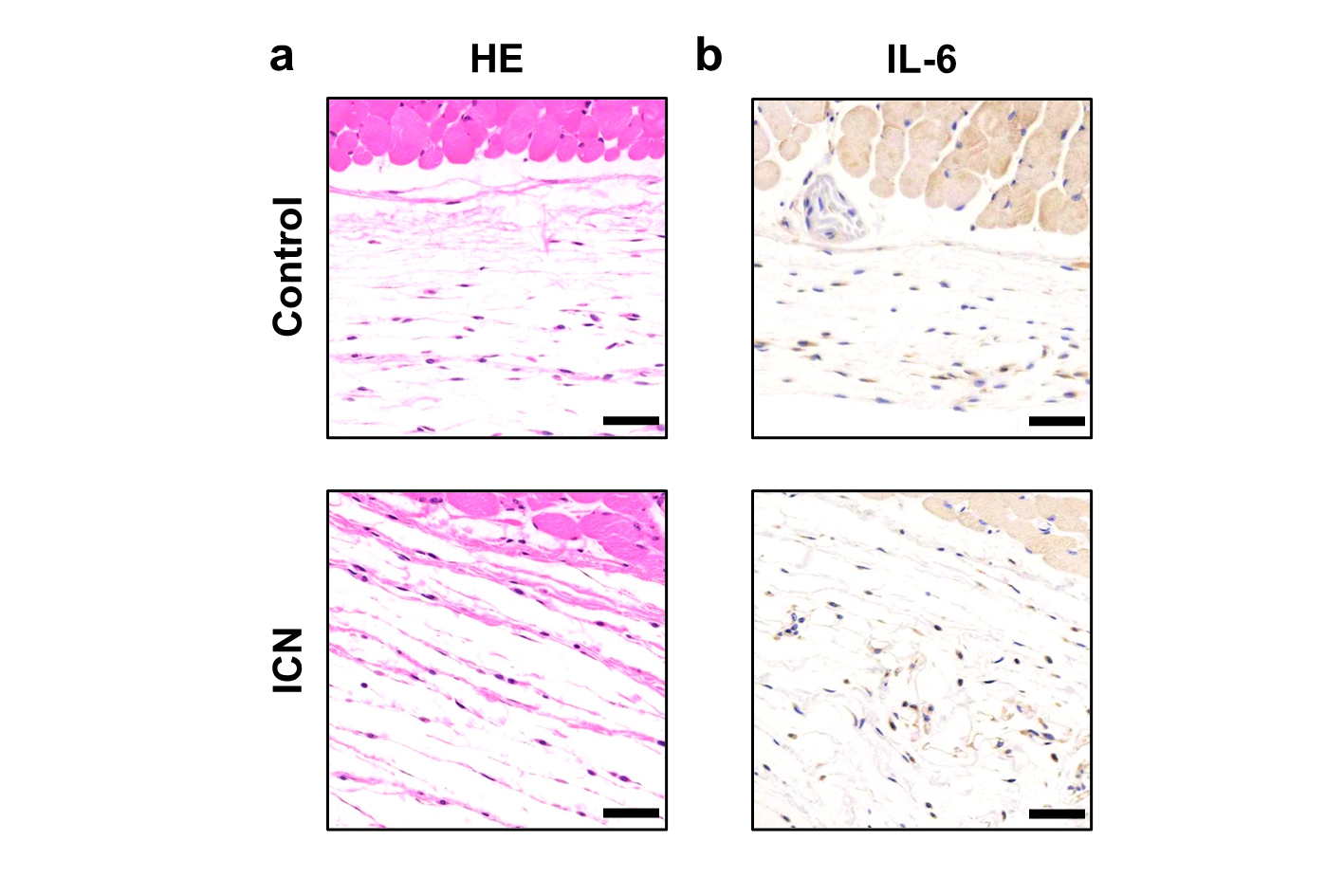


**Figure S6.** a) HE staining and b) IL-6 immunohistochemical staining after subcutaneous implantation of PBS or ICN suspension at day 14 (scale bars = 50 μm).


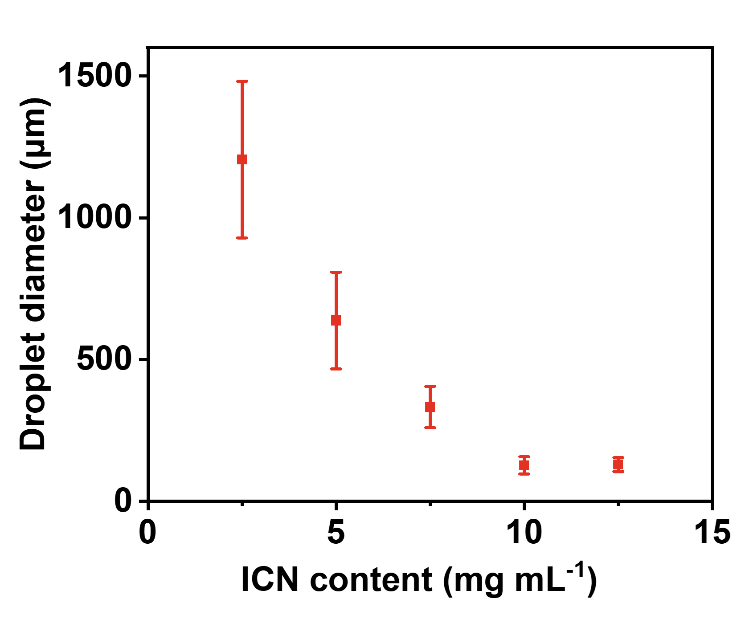


**Figure S7.** Droplet diameter of lipiodol Pickering emulsion with different ICN content.


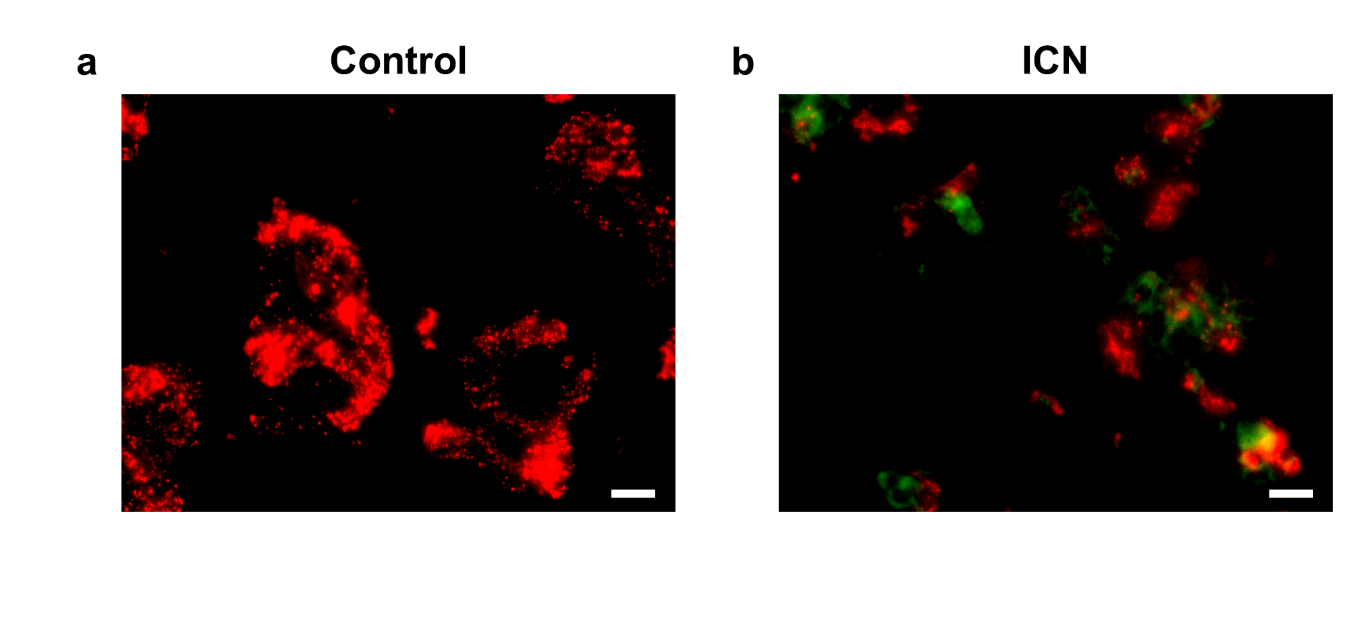


**Figure S8.** JC-1 analysis of HepG2 cells in a) the control group and b) the ICN group (scale bars = 25 μm).


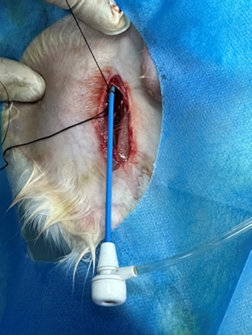


**Figure S9.** A 4F vascular sheath was implanted in the right femoral artery of a rabbit.


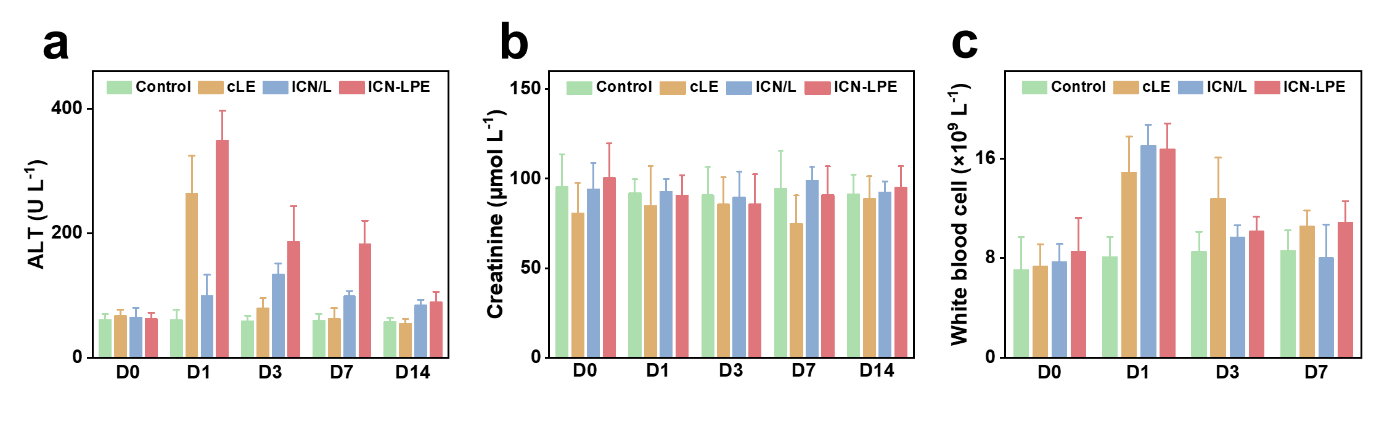


**Figure S10.** Average levels of a) alanine aminotransferase and b) creatinine in each group at days 0, 1, 3, 7, and 14 after TACE. c) Average white blood cell counts in each group at days 0, 1, 3, and 7 after TACE. The data are presented as the mean ± SD (*n* = 5).


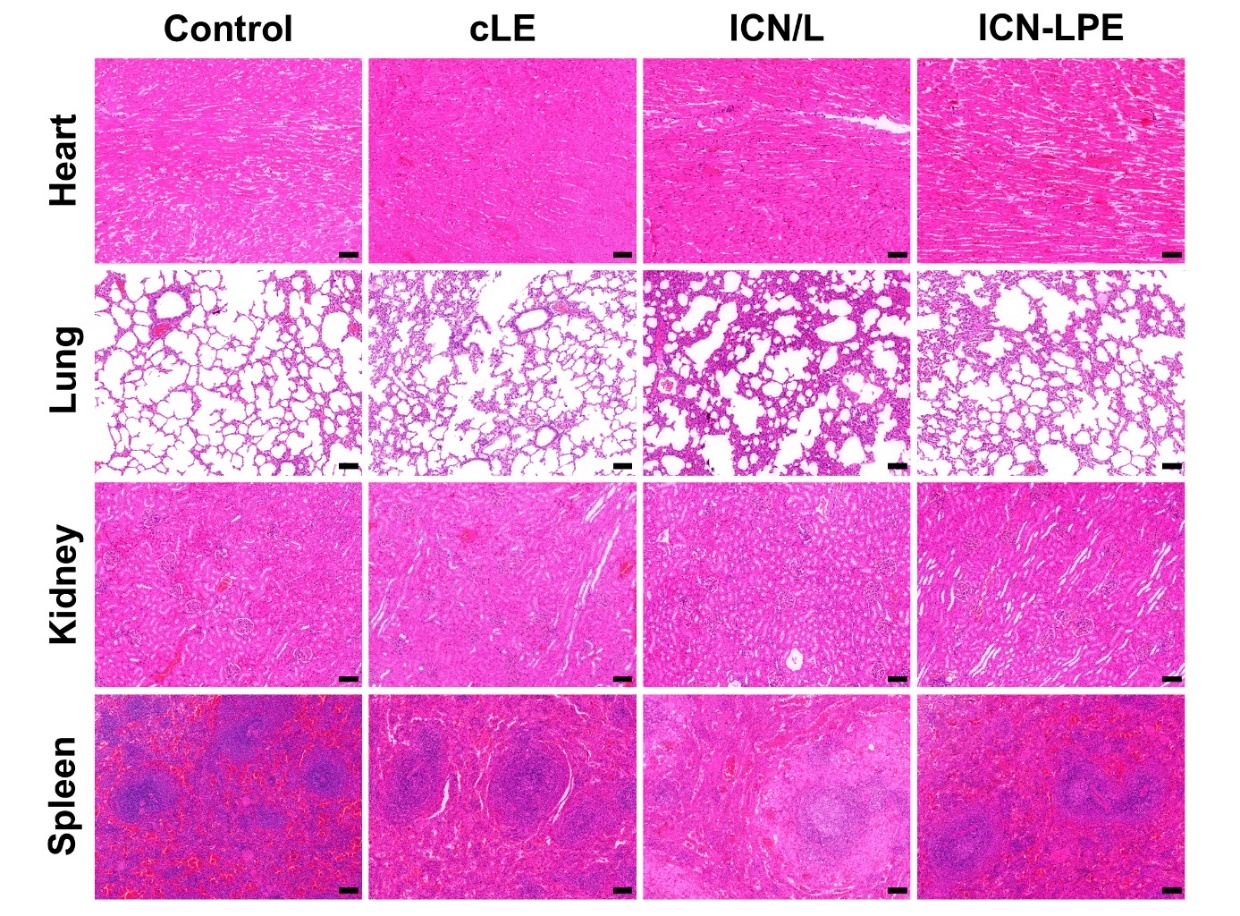


**Figure S11.** HE staining of major organs (heart, lung, kidney, and spleen) from New Zealand rabbits at day 14 after TACE (scale bars = 100 μm).

**Reference**

[1] F. Xu, Z. Tang, S. Huang, L. Chen, Y. Liang, W. Mai, H. Zhong, R. Fu, D. Wu, *Nat. Commun.* **2015**, *6*, 7221.
